# Detection of miR-155-5p and imaging lung cancer for early diagnosis: in vitro and in vivo study

**DOI:** 10.1007/s00432-020-03246-2

**Published:** 2020-05-23

**Authors:** Hai-Zhen Zhu, Chun-Ju Fang, Yi Guo, Qi Zhang, Li-Min Huang, Dong Qiu, Guang-Peng Chen, Xiu-Feng Pang, Jian-Jun Hu, Jian-Guo Sun, Zheng-Tang Chen

**Affiliations:** 1 Department of Oncology, Guizhou Provincial People’s Hospital, Guizhou Cancer Center, Guiyang, 550002 China; 2 Department of Basic Knowledge, Guiyang Nursing Vocational College, Guiyang, 400037 China; 3 Cancer Institute of PLA, Xinqiao Hospital, Army Medical University, Chongqing, 400037 China; 4 Shanghai Key Laboratory of Regulatory Biology, Institute of Biomedical Sciences and School of Life Sciences, East China Normal University, Shanghai, 200241 China; 5 Department of Pathology, Guizhou Provincial People’s Hospital, Guiyang, 550002 China

**Keywords:** Lung cancer, MicroRNA, Tumor-initiating cell, Molecular beacon, Chitosan, Molecular imaging

## Abstract

**Purpose:**

Currently, the routine screening program has insufficient capacity for the early diagnosis of lung cancer. Therefore, a type of chitosan-molecular beacon (CS-MB) probe was developed to recognize the miR-155-5p and image the lung cancer cells for the early diagnosis.

**Methods:**

Based on the molecular beacon (MB) technology and nanotechnology, the CS-MB probe was synthesized self-assembly. There are four types of cells—three kinds of animal models and one type of histopathological sections of human lung cancer were utilized as models, including A549, SPC-A1, H446 lung cancer cells, tumor-initiating cells (TICs), subcutaneous and lung xenografts mice, and lox-stop-lox(LSL) K-ras G12D transgenic mice. The transgenic mice dynamically displayed the process from normal lung tissues to atypical hyperplasia, adenoma, carcinoma in situ, and adenocarcinoma. The different miR-155-5p expression levels in these cells and models were measured by quantitative real-time polymerase chain reaction (qRT-PCR). The CS-MB probe was used to recognize the miR-155-5p and image the lung cancer cells by confocal microscopy in vitro and by living imaging system in vivo.

**Results:**

The CS-MB probe could be used to recognize the miR-155-5p and image the lung cancer cells significantly in these cells and models. The fluorescence intensity trends detected by the CS-MB probe were similar to the expression levels trends of miR-155 tested by qRT-PCR. Moreover, the fluorescence intensity showed an increasing trend with the tumor progression in the transgenic mice model, and the occurrence and development of lung cancer were dynamically monitored by the differen fluorescence intensity. In addition, the miR-155-5p in human lung cancer tissues could be detected by the miR-155-5p MB.

**Conclusion:**

Both in vivo and in vitro experiments demonstrated that the CS-MB probe could be utilized to recognize the miR-155-5p and image the lung cancer cells. It provided a novel experimental and theoretical basis for the early diagnosis of the disease. Also, the histopathological sections of human lung cancer research laid the foundation for subsequent preclinical studies. In addition, different MBs could be designed to detect other miRNAs for the early diagnosis of other tumors.

## Introduction

Owing to late-stage detection and poor treatment, lung cancer is the leading cause of cancer-related deaths worldwide (Wang et al. 2019a, b). The high mortality of lung cancer is always closely associated with late diagnosis (Zhang et al. 2019a, b). Thus, improving the early diagnosis efficiency is very important for the prognosis of patients with lung cancer. However, currently, specific and sensitive molecular targets for the early diagnosis of lung cancer are lacking (OST et al. 2014; Lipińska et al. 2019).

Tumor-initiating cells (TICs) are considered as the source of tumor occurrence, development, recurrence, and metastasis (De et al. 2018; Moro et al. 2015; Yu et al. 2016), thereby finding the TICs may be a novel breakthrough for the early diagnosis of cancers. If the TICs of lung cancer can be recognized and imaged directly based on the fluorescent substances, then the early diagnosis can be implemented. Thus, imaging of cancer cells is currently an active area of research (Lingeshwar Reddy et al. 2018). Previous studies have demonstrated that microRNAs (miRNAs, miR) extensively participate in the occurrence, development, and metastasis of cancers (Tutar et al. 2018). miRNAs are short, highly conserved, noncoding RNAs, 18–22 nucleotides in length, and can function as oncogenes or tumor suppressor genes. Thus, recognition and imaging of miRNAs related to lung cancer cells or TICs might constitute a critical strategy for the early diagnosis of the disease. Among these, miR-155 plays a major role in the occurrence, development, and diagnosis of lung cancer (Zhang et al. 2018; Shao et al. 2019).

Molecular beacon (MB) is a stem loop-structured (hairpin) oligonucleotide that has a reporter fluorophore and a quencher. MB cannot produce fluorescence; however, it hybridizes with the target DNA, RNA, or miRNA to produce fluorescence signals. Therefore, real-time imaging and quantitative analysis of target DNA, RNA, and miRNA can be implemented by detecting the fluorescence signal and intensity (Zhang et al. 2017; Moon et al. 2019; Kang et al. 2011). Since MB is an oligonucleotide, it cannot traverse the cell membrane easily due to the negative surface charge, and is degraded by endogenous nucleases, and, hence, cannot enter the cells directly but only when delivered by an ideal carrier (Kim et al. 2015; Dong et al. 2011). Chitosan (CS) is a natural cationic polysaccharide and has been widely used to mediate the gene transfection of plasmids and small interfering RNAs (siRNAs) (Cao et al. 2019; Rahmani et al. 2015; Jaiswal et al. 2019). In a previous study (Zhu et al. 2014), we used CS nanoparticles combined with MB for the detection and imaging miR-155-5p in non-small cells lung cancer.

**Fig. 1:**
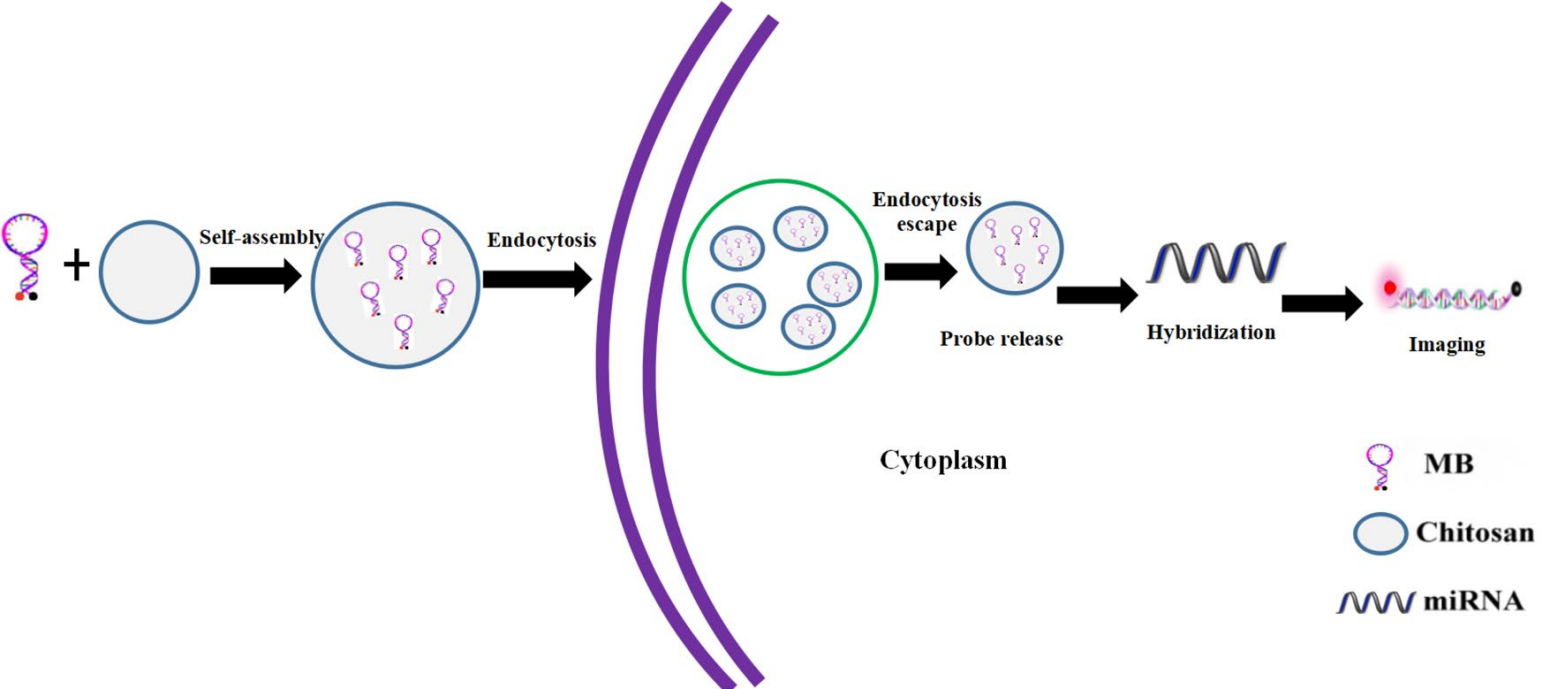
Schematic showing the delivery of miR-155-5p MB into the cells via CS nanoparticles for the detection and imaging of miRNA

The occurrence of lung adenocarcinoma of lox–stop–lox (LSL) K-ras G12D transgenic mice can be induced by instilling Cre adenovirus into the nasal cavity after adulthood. The transgenic mice model displays the dynamic process from normal lung tissues to atypical hyperplasia, adenoma, carcinoma in situ, and adenocarcinoma, and simulates the occurrence and development of human lung adenocarcinoma (DuPage et al. 2009; Sutherland et al. 2014), thereby serving as a preferred animal model for the early diagnosis of lung cancer. Therefore, in the present study, nanotechnology and MB technology were combined, and lung cancer cells, TICs, subcutaneous and lung xenografts in nude mice, and transgenic mice were used as the study subjects to investigate the feasibility of CS nanoparticles (as miR-155-5p MB carrier) in recognizing and imaging miR-155-5p in lung cancer cells (Fig. 1). This strategy would provide a novel technology for the early diagnosis of lung cancer.

## Materials and methods

### Cell culture

Human lung adenocarcinoma cells A549 and human small cell lung cancer cells (SCLC) H446 were purchased from American Type Culture Collection (Manassas, VA, USA). Human lung adenocarcinoma cells SPC-A1 were purchased from the Shanghai Institute of Cellular Biology. A549, SPC-A1, and H446 lung cancer cell lines were cultured in an incubator with 5% CO_2_ at 37 °C using RPMI 1640 medium (Hyclone, Waltham, MA, USA) containing 10% fetal bovine serum (Haoyang, Tianjin, China). According to the previous study (Yao et al. 2014), TICs (CD133^+^CD338^+^ cells) were isolated from A549 cells as described previously and cultured in serum-free DMEM/F12 (Hyclone, Waltham, MA, USA) medium.

### Animal model establishment

All in vivo experiments were approved by the Xinqiao Hospital Animal Care and Use Committee. Under aseptic experimental conditions, 1 × 10^6^ A549 and H446 cells were subcutaneously injected into the left back or front of anesthetized 6–8-week nude mice to establish subcutaneous xenograft models of lung adenocarcinoma and small cell lung cancer. 1 × 10^6^ A549 and H446 cells were injected via the tail veins to establish lung xenograft models. Thirty days following injection of lung cancer cells, subcutaneous transplanted tumors (Vol ~ 300 mm^3^) and lung transplanted tumors appeared in all mice. Cre adenovirus (Hanbio, Shenzhen, China), with the titer of 5 × 10^9^ PFU that can secrete Cre enzyme, was slowly instilled into the nasal cavity of the transgenic mice at the age of 8 weeks. These mice were sacrificed at weeks 4, 6, 8, and 12 after instilling adenovirus. One side of the lung tissues in the transgenic mice at different disease stages and the lung xenograft models were resected, fixed in 4% paraformaldehyde (Boster, Wuhan, China), and sliced as 4 μm paraffin sections for HE (hematoxylin–eosin) staining to observe the different pathological changes. The subcutaneous xenografts and lung xenografts from the other side were frozen in the liquid nitrogen for detecting miR-155-5p expression.

### Confocal microscopy

Based on a previous study (Zhu et al. 2014), CS nanoparticles (Guanghan Hengyu, Sichuan, China), hsa-miR-155-5p MB, mmu-miR-155-5p MB, or random sequence MB (RS MB) (Sangon, Shanghai, China) were mixed at the ratio of Wcs/W_MB_ = 7:1 to synthesize the CS-MB nanoparticles. Hsa-miR-155-5p MB (5′-Cy5-C + CAGCG-ACC + CCT + ATCA + CGAT + TAGCATTAA-CGCT + GG-BHQ3-3′) and Mmu-miR-155-5pMB (5′-Cy5-C + CAGCG-ACC + CCT + ATCA + CAAT + TAGCATTAA-CGCT + GG-BHQ3-3′) were designed according to the miR-155-5p sequences. A random sequence MB (RS MB: 5′-Cy5-C + CAGCG-AC + GCCA + ATG + ACC + TTA + AGCATTAA-CGCT + GG-BHQ3-3′) complementary to no known gene sequence was used as a negative control. The MB had a Cy5-molecule attached to the 5′-end and a black hole quencher-3(BHQ3) attached to the 3′-end. The underlining bases were the ones added to form a stem, and the +N represented the LNAs (locked nucleic acids) modified bases. All the synthetic oligodeoxynucleotides were purchased from Sangon Company (Shanghai, China). 1 × 10^4^ A549, SPC-A1, H446, and TIC lung cancer cells were seeded in culture dishes for 24 h. Subsequently, 200 μL of mixed CS-MB nanoparticles were added into the culture dishes, and 100 μL Opti-MEM^®^ I reduced serum medium (Life Technologies, Waltham, MA, USA) was added to adjust the concentration; and the final concentration of MB was 200 nmol/L. RS MB was used as a negative control. These cells were incubated in a CO_2_ incubator at 37 °C for 2 h, followed by PBS washes. Then, the nuclei were stained with Hoechst 33342 (Beyotime, Shanghai, China) at 37 °C for 20 min, followed by imaging using confocal microscopy (Leica TCS Confocal Microscope, Leica, Wetzlar, Germany). An equivalent of 1 mL cell lysate (Beyotime, Shanghai, China) was added into each culture dish for cell lysis. A volume of 100 ml suspension was distributed into each of 6-wells of a 96-well plate, and the fluorescence intensity of Cy5 (excitation 649 nm/emission 670 nm) per well was detected by Varioskan Flash (Thermo, Waltham, MA, USA) in each group.

### Quantitative RT-PCR

Total RNA of cells and tissues was extracted from A549, SPC-A1, H446 cells, and TICs, as well as subcutaneous xenografts, lung xenografts, and lung tissues of transgenic mice at different stages of the disease using RNAiso plus TRIzol reagent (TaKaRa, Japan). One microgram total RNA was used for cDNA synthesis,reverse transcription was performed with the PrimeScript RT reagent kit (Takara, Japan) at 37 °C for 15 min and 85 °C for 5 s. The changes in the expression of miR-155-5p in the four types of cells, xenografts, and transgenic mice lung tissues were detected using the SYBR PCR Master Mix reagent kits (TaKaRa, Japan). The PCR conditions for the miR-155 and U6 genes were: pre-denaturation at 95 °C for 30 s, followed by 40 cycles of denaturation 95 °C for 5 s and 60 °C for 34 s. For each sample, three duplicated wells were used for miRNA detection. U6 served as the internal reference gene and has-miR-155-5p or mmu-miR-155-5p as the target gene. The relative expression of miR-155-5p was evaluated using the ∆∆Ct method. All the premier sequences were synthesized by RiboBio Company (Riobio, Guangzhou, China). The hsa-miR-155-5p, mmu-miR-155-5p, and U6 Primer Set catalog numbers’ are MQPS0000685, MQPS0002476, and MQPS0000002, respectively.

### In vivo detection of miR-155-5p and imaging of the cancer cells

After the mice were anesthetized by intraperitoneal injection of 4% chloral hydrate (Chron Chemicals, Chengdu, China), an equivalent of 100 μL of CS-miR-155-5p MB or CS-RS MB nanoparticles was injected via the tail veins. The final concentration of MB was 2 μM. There are two negative control groups, in the subcutaneous and lung xenograft nude mice models; A549 subcutaneous and lung xenografts models were used as the negative control groups after the injection of CS-RS MB; in the transgenic mice model, mice without intranasal inhalation of the adenovirus after the injection of CS-miR-155-5p MB were used as the negative control group. Depilatory cream was used for removing the black hair on the thorax of the transgenic mice for detection of Cy5 fluorescence signal. After 2 h, the mice were placed on the in vivo imaging system (IVIS) platform, and the fluorescence signals from subcutaneous xenografts, lung xenografts, and transgenic mice lung tissues were imaged and detected by the IVIS Spectrum Imaging System (Caliper Life Sciences, Boston, USA). After in vivo imaging, the mice were sacrificed by dislocating the neck. The xenografts and lung tissues were removed and imaged again by the IVIS Spectrum Imaging System. Next, the frozen sections were prepared and then fixed in paraformaldehyde, followed by DAPI (Beyotime, Shanghai, China) staining of the nucleus. After that, the fluorescent images were photographed by confocal microscopy.

### Detection and imaging of miR-155-5p in the human lung cancer tissues

This study was approved by the Medical Ethics Committee of Xinqiao Hospital (NO.2017004-011), and all participants were completely informed and signed the written informed consents. As described previously (Peng et al. 2005), three cases of fresh lung squamous cell carcinoma specimens and three cases of adenocarcinoma specimens were collected from the XinQiao Hospital of Army Medical University after confirmed by postoperative pathology. Frozen sections were prepared and fixed in ice acetone (Chron Chemicals, Chengdu, China) for 10 min. Then, 50 μL hsa-miR-155-5p MB or RS MB at 100 nmol/L was dropped to each section, followed by incubation at 37 °C for 60 min. Subsequently, the nuclei were stained by DAPI. The fluorescence signal of miR-155 in the lung tissues was detected by confocal microscopy; RS MB was used as the negative control.

### Statistical analysis

An independent sample *t* test was performed for comparison between the two groups, and one-way ANOVA was performed for comparison among multiple groups. *P *< 0.05 was considered statistically significant. All data were represented as means ± standard deviations.

## Results

### Fluorescence imaging of cancer cells in vitro

**Fig. 2:**
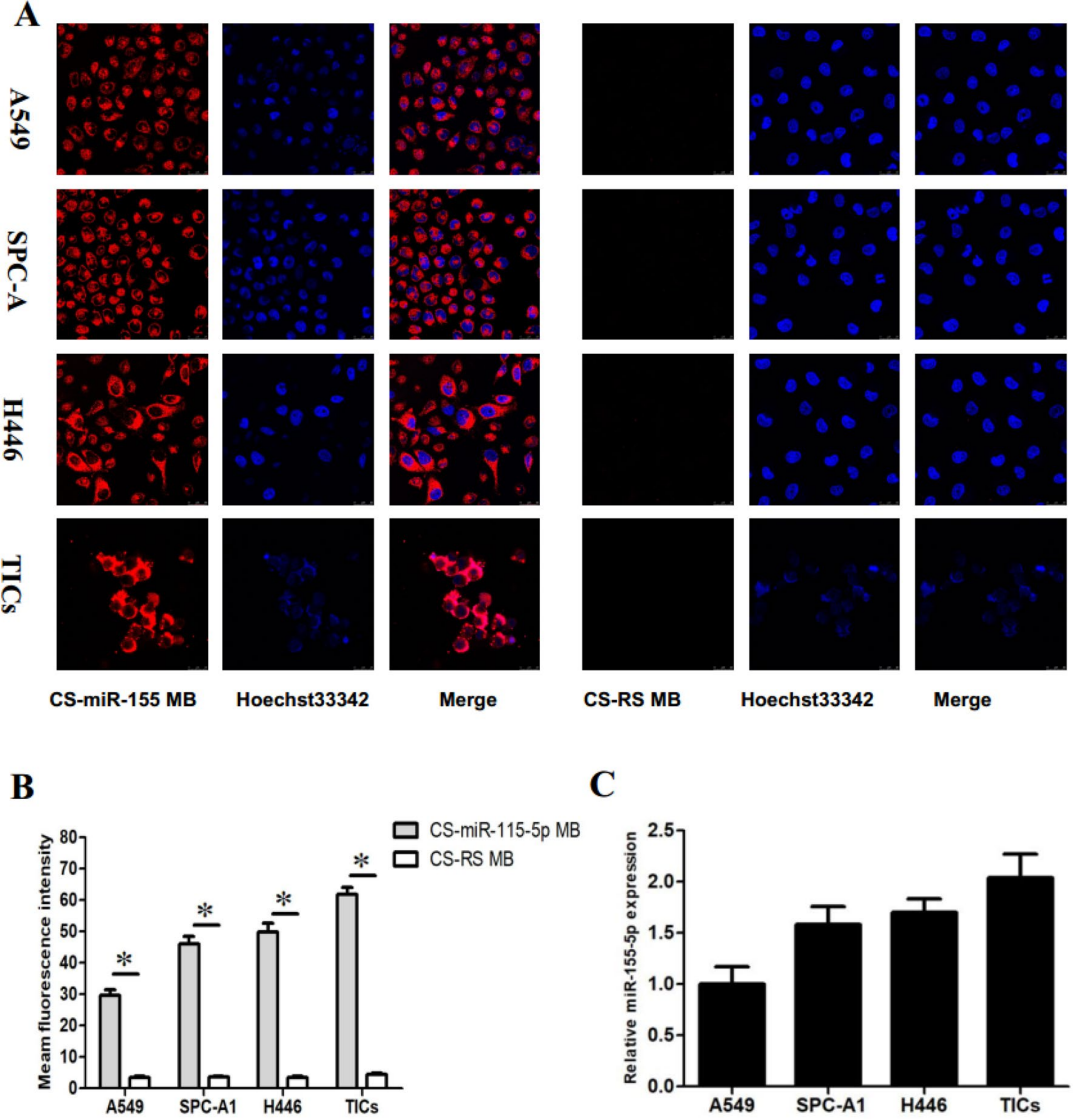
Fluorescence imaging and detection in viable cancer cells. **a** Confocal microscopy imaging of the four cells after delivery of the miR-155-5p MB (red) by CS nanoparticles. RS MB was used as a negative control. The cell nucleuses were stained by Hoechst33342 (blue). Scale bar = 25 μm. **b** Fluorescence intensity of Cy5 was measured after imaging (*n* = 6) (*p < 0.05). **c** Relative miR-155-5p expression was detected in A549, SPC-A1, H446 cells, and TICs by qRT-PCR

To determine whether the CS-MB probe could detect the level of miRNA expression in viable cells, their ability to detect target microRNAs was assessed. In vitro experiments showed strong red fluorescent signals in the cytoplasm in each group of cells in the presence of CS-miR-155 MB, while only a few signals were detected in the nuclei (Fig. 2a). However, no significant red fluorescent signals were detected in the CS-RS MB group (Fig. 2b). Furthermore, the average fluorescence intensity of the 6 wells in each group was calculated. The mean fluorescence intensity of TICs was the strongest, followed by H446, SPC-A1, and A549 cells, and the fluorescence intensity was significantly increased in the presence of the CS-miR-155-5p MB compare with the presence of the CS-RS MB (Fig. 2c). Moreover, the fluorescence intensity trend of the four cells, after miR-155-5p MB was transfected using CS nanoparticles, was similar to the expression of miR-155-5p detected by qRT-PCR (Fig. 2c). Therefore, CS-MB probe could be used for detecting miR-155-5p expression and imaging lung cancer cells.

### HE staining and miR-155-5p expression in xenografts and transgenic mice models

**Fig. 3:**
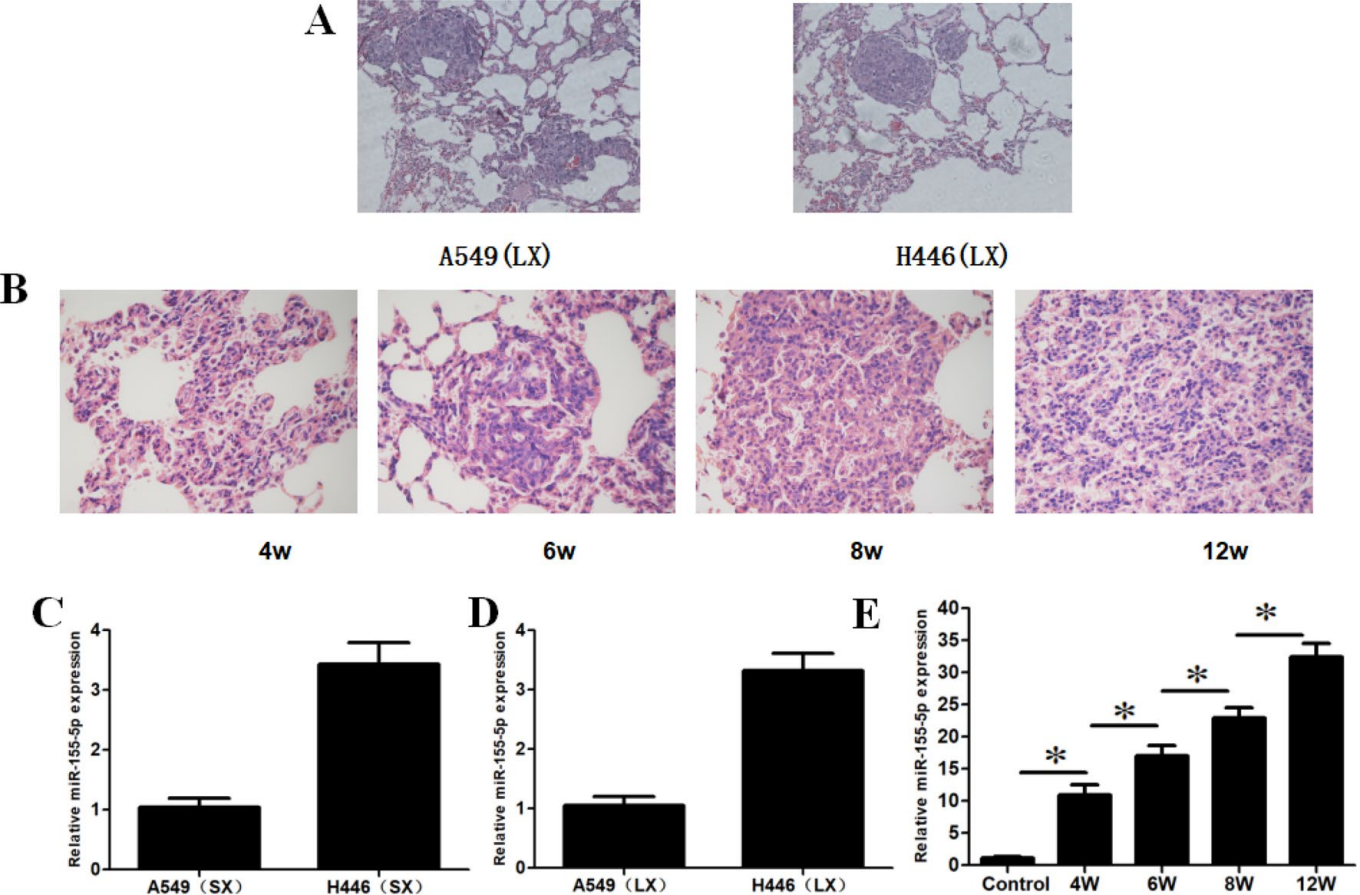
Establishment of animal models and detection of miR-155-5p expression. **a** HE staining in A549 and H446 lung xenograft(LX)models (× 200). **b** HE staining at 4, 6, 8, and 12 weeks in transgenic mice models after instillation of adenovirus (× 400). **c**, **d** miR-155-5p expression in the subcutaneous xenografts (SX) and lung xenografts (LX) of nude mice models (*n* = 8). **e** miR-155-5p expression in transgenic mice at different disease stages (*n* = 8) (*p < 0.05)

HE staining was used to prove that lung xenograft model and different disease stages of transgenic mice were established, while qRT-PCR technology was used to detect the expression of miR-155-5p in lung cancer cells and animal models. HE staining revealed that lung adenocarcinoma cells A549 and small cell lung cancer cells H446 planting nodules were implanted in the lung tissues of nude mice (Fig. 3a), indicating successfully establishment of lung xenograft models. At 4, 6, 8, and 12 weeks after instillation of adenovirus, different disease processes of atypical hyperplasia, adenoma, carcinoma in situ, and lung adenocarcinoma could be observed in lung tissues of the transgenic mice (Fig. 3B), suggesting that lung cancer models of different disease stages were established successfully. qRT-PCR showed that miR-155-5p expressed in subcutaneous xenografts (SX) and lung xenografts (LX) of nude mice (Fig. 3c, d) as well as lung tissues of transgenic mice at different stages of the disease. In transgenic mice, miR-155-5p expression increased significantly with the progression of lung cancer as compared to that at 4, 6, 8, and 12 weeks (Fig. 3e), suggesting that miR-155-5p plays a major role in the occurrence and development of lung cancer, and can be used as a target for tracing, detection, and imaging.

### Fluorescence imaging of cancer cells in xenografts models

**Fig. 4:**
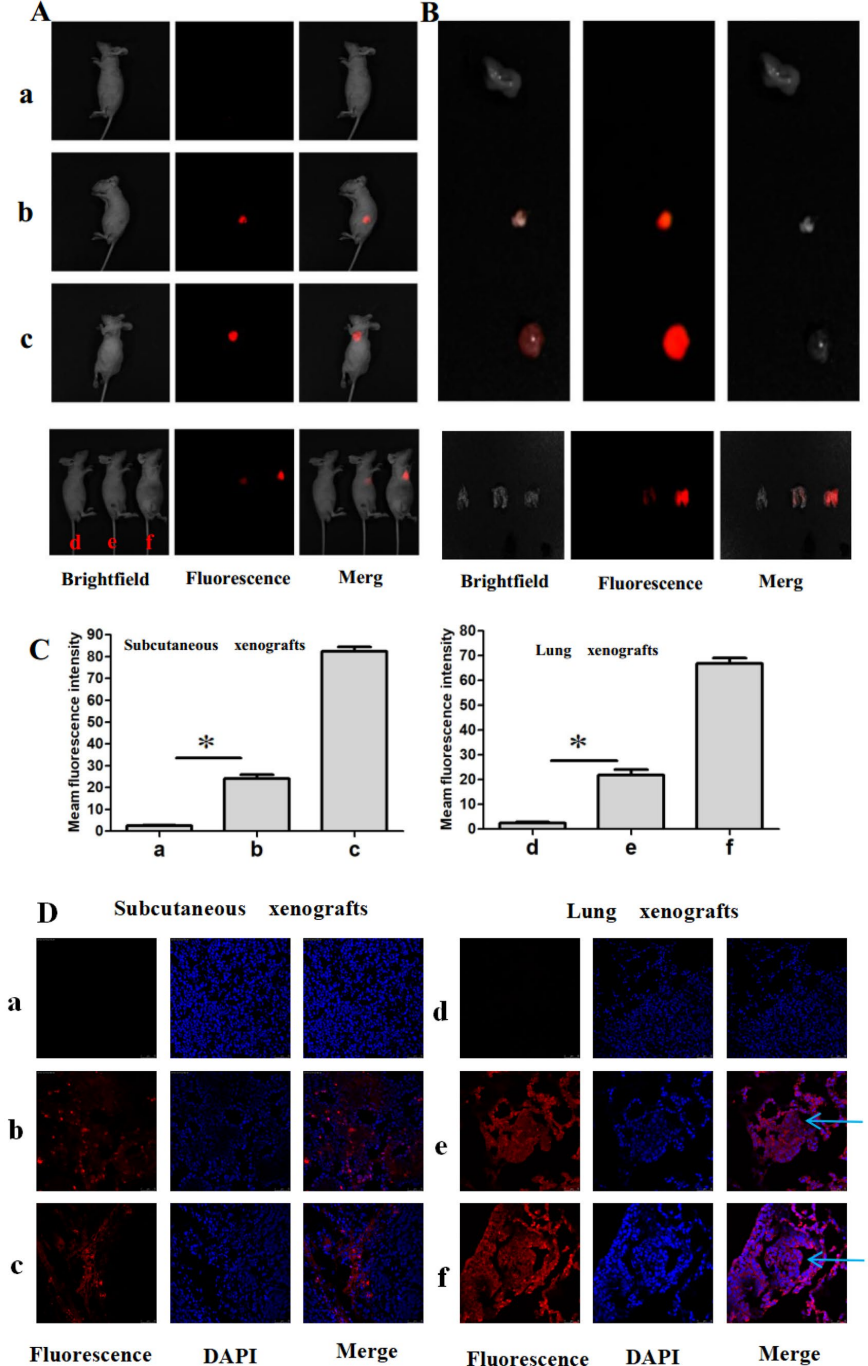
In vivo identification of miR-155-5p and fluorescence imaging of cancer cells in xenografts models. **a** Imaging the subcutaneous and lung xenografts after injection of CS-MB via the tail veins. Subcutaneous xenografts model, a: A549 treated with CS-RS MB, b:A549 treated with CS-miR-155-5p MB, c: H446 treated with CS-miR-155-5p MB. Lung xenografts model, d: A549 treated with CS-RS MB, e:A549 treated with CS-miR-155-5p MB, f: H446 treated with CS-miR-155-5p MB. Groups a and d were used as the negative controls. **b** Imaging the xenografts after removal. **c** Fluorescence intensity was analyzed after injection (*n* = 8) (*p < 0.05). **d** Confocal microscopy imaging of the xenografts tissues after transfection with CS-miR155-5p MB or CS-RS MB (red). Cell nuclei were stained by DAPI (blue). Scale bar 25, 50 μm. Arrow: planting nodule and cancer cells

The ability of CS-MB probe to detect miRNA in vivo was further investigated in xenografts models. The in vivo imaging showed fluorescent signals with varying intensities were detected in the subcutaneous xenograft and chests (Fig. 4a). After the tumor or lung tissues were removed, the imaging showed that the fluorescent signals were stronger in the H446 group than in the A546 group. The distribution of the fluorescent signals was found to be consistent with the size of subcutaneous xenografts (Fig. 4b). The fluorescence intensity was analyzed after injection using the Spectrum Living Image 4.0 software, and the results demonstrated that fluorescent signals in the H446 group with high miR-155-5p expression were stronger than those in the A549 group. However, no fluorescent signals were detected in the CS-RS MB negative control group of the A549 SX and LX models (Fig. 4c). Interestingly, the fluorescence intensity trend was similar to the expression of miR-155-5p detected by qRT-PCR (Fig. 3c, d). The frozen sections were prepared using tumor tissues and lung tissues and examined by confocal microscopy, which showed that the fluorescent signals were from some tumor cells in the subcutaneous xenografts model. In lung xenografts model, the fluorescent signals were from some tumor cells and some alveolar epithelial cells. Compared to the A549 group, the fluorescent signals were brighter in the H446 group (Fig. 4d). Moreover, the intense fluorescent signals in the cytoplasm suggested that CS nanoparticles could be used as carriers to transport MB in vivo, thereby facilitating its entry into the cytoplasm to specifically bind to miR-155 and produce the signals.

### Fluorescence imaging cancer cells in transgenic mice models

**Fig. 5:**
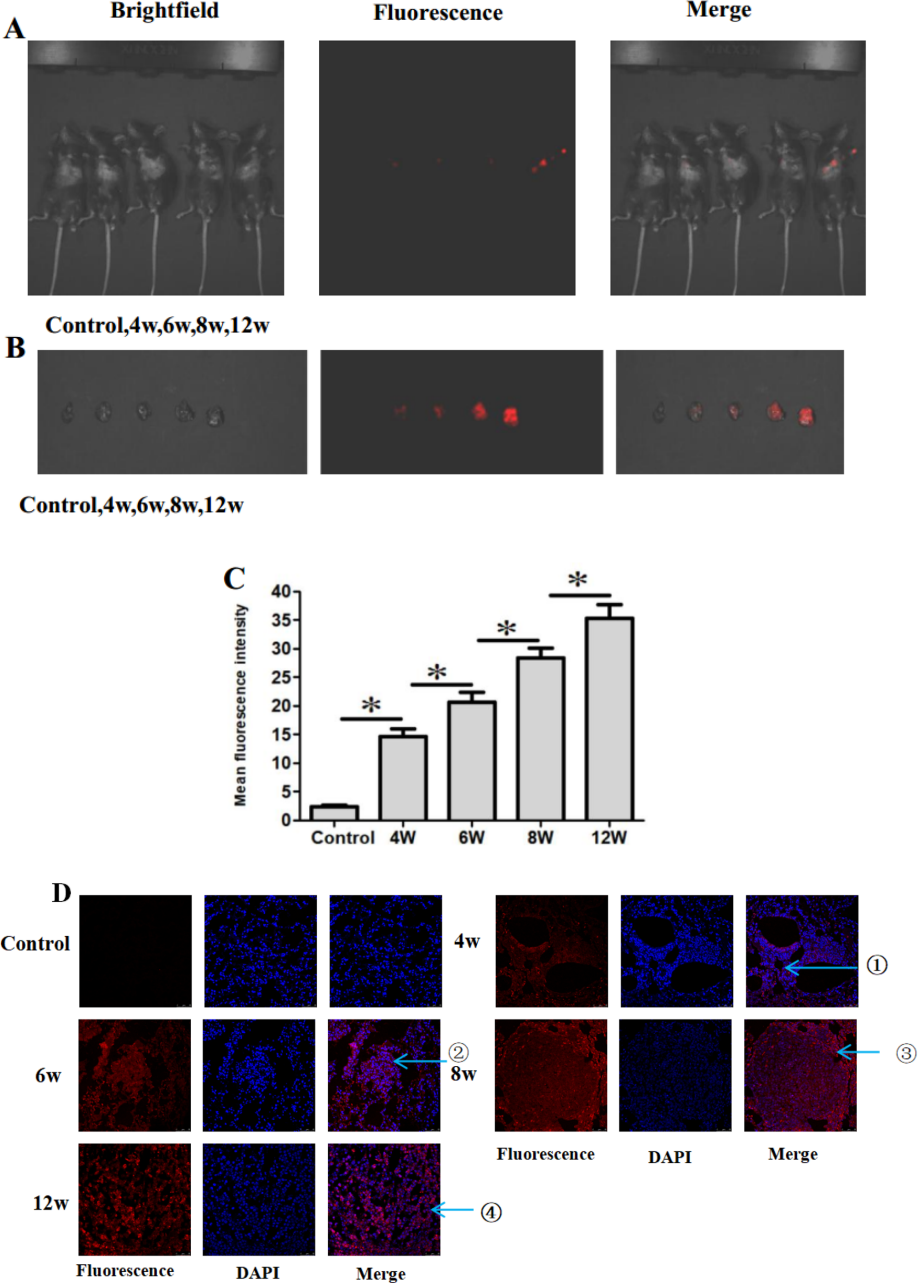
In vivo identification of miR-155-5p and fluorescence imaging of cancer cells in transgenic mice at different stages of the disease. **a** Imaging the lung after injection of CS-miR-155-5p MB nanoparticles via the tail vein. Mice without intranasal inhalation of the adenovirus were used as the control group. **b** Imaging the lungs after removal. **c** Fluorescence intensity was analyzed after injection (*n* = 8) (*p < 0.05). **d** Confocal microscopy imaging of the different pathological changes after transfection with CS-miR-155-5p MB. Cell nuclei were stained by DAPI (blue). Scale bar 50 μm. Arrow ①: atypical hyperplasia. Arrow ②: adenoma. Arrow③: carcinoma in situ. Arrow ④: adenocarcinoma

The ability of CS-MB nanoparticles to detect miR-155-5p at the different disease stages in the transgenic mice was further investigated. After CS-MB nanoparticles were injected via the tail veins using the same method, fluorescent signals of varying intensities could be detected in the lung tissues of transgenic mice (Fig. 5a). Also, the fluorescent signals were detected at different disease stages after removal of the lung tissue (Fig. 5b). Analysis of the fluorescence intensity showed an increasing trend with the progression of the disease (Fig. 5c). However, the fluorescence signals were not detected in the control group of mice without intranasal inhalation of the adenovirus. It was similar to the expression of miR-155 detected by qRT-PCR (Fig. 3e). Confocal microscopy of the frozen sections of the lung tissue showed that fluorescent signals were from the pleomorphic or cancer cells and some alveolar epithelial cells (Fig. 5d). The occurrence and development of lung cancer were dynamically monitored by detecting varying fluorescence intensities, which provided a novel technology for the early diagnosis of lung cancer.

### Fluorescence imaging and identification of miR-155 in human lung cancer tissues

**Fig. 6:**
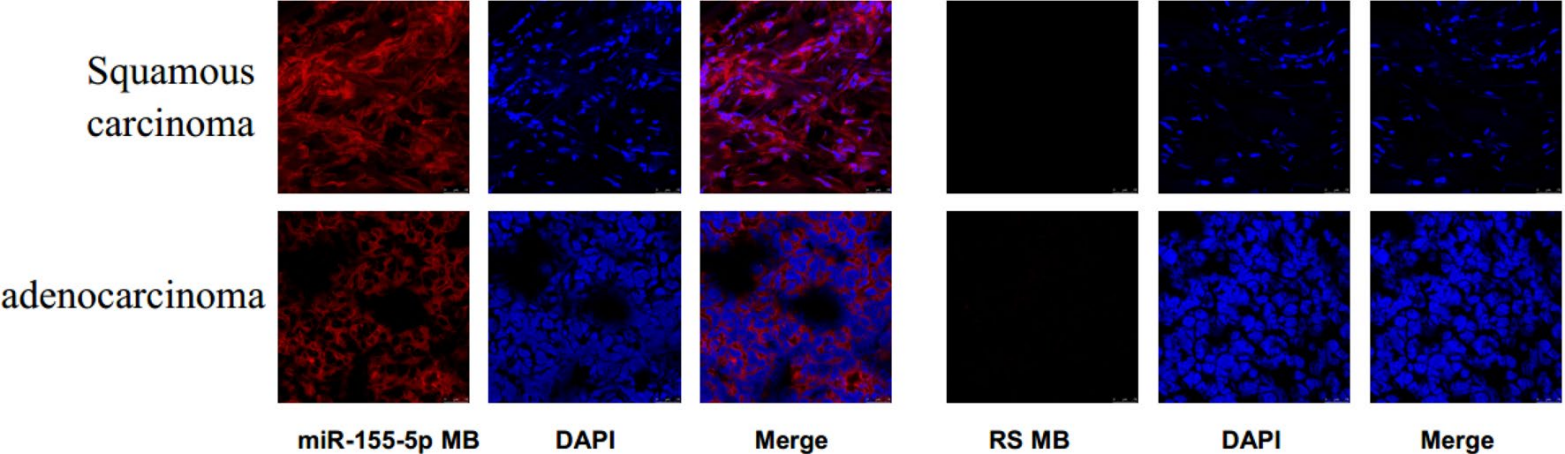
Identification of miR-155-5p and fluorescence imaging of the cancer cells in human lung squamous carcinoma and adenocarcinoma tissues. RS MB was used as a negative control. Scale bar 50 μm

The ability of miR-155-5p MB to detect miR-155-5p in human lung cancer tissues was investigated further. After miR-155-5p MB was added to frozen lung cancer tissues, red fluorescent signals with different intensities could be detected in squamous and adenocarcinoma tissues. The majority of the red fluorescent signals were detected in the cytoplasm of the tumor. However, no significant fluorescent signals were detected in the negative RS MB group (Fig. 6). These findings suggested that miR-155-5p MB can bind to miR-155-5p in the tumor tissues, leading to fluorescent signals, which laid a foundation for subsequent preclinical study.

## Discussion

Previous studies have demonstrated that miR-155 exerts a cancer-promoting effect in the occurrence and development of multiple cancers. miR-155 is not only overexpressed in several cancers such as breast, colorectal, gastric, and liver cancers, but also increased abnormally in the tissues and serum of the lung cancer patients, thereby miR-155 is a potential molecular marker for the early diagnosis of lung cancer, Moreover, lung cancer patients with high expression of miR-155 have a short survival time and poor prognosis (Liu et al. 2017; Zhu et al. 2018). MB technology can implement a convenient, rapid, and dynamic detection of miRNA. The detection of genes using MB is a highly sensitive and specific method, and can detect the difference of one base. This technology facilitates the detection of intracellular genetic mutations and changes in the expression of intracellular genes (Zhang et al. 2019a, b; Mahani et al. 2019).

Since MB is an oligonucleotide, it needs to be carried into live cells by ideal vectors. The optimal vectors not only protect the MB from degradation but also mediate target recognition of nucleic acid sequences in the cells, thereby facilitating the visualization study of live cells. CS has low toxicity and immunogenicity and can be obtained in a large quantity from the natural environment (Hejjaji et al. 2019). Due to its polycationic characteristics, chitosan can form complexes with negatively charged nucleic acids through electrostatic interactions, leading to the condensation and protection of nucleic acids. Chitosan has been regarded as a highly attractive biopolymer to deliver nucleic acids intracellularly and induce a transgenic response, so the CS nanoparticles were used as biomaterial carrier for MB delivery and miRNA detection in living cells (Santos-Carballal et al. 2018; Chuan et al. 2019).

Accumulating evidence has shown that TICs are the source of the occurrence, development, and recurrence of all tumors. Thus, finding and imaging TICs could provide a novel method for the early diagnosis of lung cancer (Peiffer et al. 2019). Therefore, in this study, lung adenocarcinoma cell lines A549 and SPC-A1, SCLC line H446, and TICs with high expression of miR-155-5p were used as models. After miR-155-5p MB was transfected with CS nanoparticles, the results showed that miR-155-5p MB could detect miR-155-5p and image the lung cancer living cells. Moreover, the fluorescence intensity of TICs with the highest expression of miR-155-5p was the maximal and can be easily detected. Recognized and imaged the TICs of lung cancer would be a method for the early diagnosis of lung cancer. Further analysis of the fluorescence intensity demonstrated that the fluorescence intensity of the four cell lines after transfection with MB was consistent with the expression of miR-155-5p as assessed by qRT-PCR. This phenomenon indicated that miR-155-5p MB designed in our study could dynamically monitor the changes in the expression of miR-155-5p in lung cancer living cells with high sensitivity. These findings demonstrated that miR-155-5p in living cells and TICs of lung cancer could be recognized and imaged at the cell level. Thus, the present study provided a new theoretical basis for searching lung cancer cells or tumor-initiating cells and applying them in the diagnosis of lung cancer.

At the animal level, after CS-MB nanoparticles were injected into xenograft and transgenic mouse models via the tail veins, strong or weak fluorescent signals could be detected in the tumor tissues. The endocytosis of CS-MB nanoparticles facilitates the entry and accumulation of MB in the tumor cells to identify microRNA, thus producing strong fluorescent signals, which could facilitate the early diagnosis of lung cancer by recognizing the highly expressed miR-155-5p and imaging the cancer cells. However, the fluorescent signals were detected in some alveolar epithelial cells, the specificity of the probe should be further investigated. In the other hand, dynamic monitoring of the occurrence and development of lung cancer based on the difference in the fluorescence intensity in transgenic mice models provided a novel method for the early diagnosis of the disease.

Several studies have demonstrated that miR-155 is highly expressed in the lung cancer tissues as compared to the normal lung tissues (Mohamed, et al. 2018). In the current study, tissue specimens from three cases of clinical lung squamous cell carcinoma and three cases of adenocarcinoma were used. The results showed that red fluorescent signals with different intensities could be detected after frozen lung cancer tissues were incubated with miR-155-5p MB, indicating that miR-155-5p MB can bind to miR-155-5p in the tissues to produce fluorescent signals, which laid the foundation for subsequent preclinical studies.

In the current study, both in vivo and in vitro experiments showed that CS could be used as an ideal carrier of MB for entry into the cells. It can recognize the highly expressed miR-155-5p and image the cancer cells of TICs, lung cancer xenografts, and transgenic mice in the different stages of the disease. Furthermore, CS-MB nanoparticles could dynamically monitor the occurrence and development of lung cancer by detecting the difference in the fluorescence intensity. In addition, MB was used to primarily recognize and image miR-155-5p in human lung cancer tissues. Thus, this study provided a novel experimental basis for the early diagnosis of lung cancer and laid a foundation for subsequent preclinical studies. In addition, different MBs could be designed as targeted diagnostic probes to detect other highly expressed miRNAs in many different cancers or TICs.
